# Assessing the impact of COVID-19 pandemic on all-cause mortality and child mortality in a population cohort of Iganga Mayuge HDSS in Eastern Uganda (2015–2021)

**DOI:** 10.1186/s12963-025-00435-4

**Published:** 2025-12-08

**Authors:** Dan Kajungu, Betty Nabukeera, Jean Bashingwa, Chodziwadziwa Kabudula, Beth. T. Barr, Donald Ndyomugyenyi, Akello Mercy Consolate, Collins Gyezaho, Elizeus Rutebemberwa

**Affiliations:** 1 Makerere University Centre for Health and Population Research (MUCHAP), Iganga Mayuge Health and Demographic Surveillance Site (IMHDSS), Kampala, Uganda; 2 SAMRC/Wits Rural Public Health and Health Transitions Research Unit (Agincourt), Faculty of Health Sciences, School of Public Health, University of the Witwatersrand, Johannesburg, South Africa; 3 Department of Health Policy, Planning and Management, Makerere University School of Public Health, Kampala, Uganda; 4 Division of Epidemiology and Biostatistics, Department of Global Health, Faculty of Medicine and Health Sciences, Stellenbosch University, Stellenbosch, South Africa; 5 Nyanja Health Research Institute, Lilongwe, Malawi

**Keywords:** Excess mortality, COVID-19, Under-five mortality, Health and demographic surveillance system (HDSS), Uganda

## Abstract

**Background:**

Efforts to track the mortality and public health impact of the coronavirus disease (COVID-19) in Uganda have been hampered by weak Civil registration and vital statistics (CRVS) system and suboptimal health seeking behaviors or patterns. Evaluating unexplained increases in all-cause mortality provides a complete picture of the impact of COVID-19 pandemic and guide public health policies and resource allocation to protect the most vulnerable populations.

**Methods:**

The longitudinal population cohort data on demographic events and socioeconomic status collected from 2015 to 2021 within the Iganga Mayuge Health and Demographic Surveillance System (IMHDSS) was used. Number of deaths and person years at risk were counted for each quarter of the year from January 2015 to December 2021 and classified as “pre-pandemic” (before January 2020), and “during pandemic” (January 2020 to December 2021). Crude mortality rates were computed comparing the two periods. Time series model was used to estimate excess mortality and to locate the exact time when excess deaths occurred. Cox Proportional Hazard model was used to estimate the Hazard ratio associated with death.

**Results:**

A total of 132,367 individuals were followed up from 2015 to 2021 and 3,424 deaths were registered. Slightly more than a half of all deaths (53%, n = 1,827) were male, and 65.4% (n = 2,238) were rural residents. Children under five years had a significantly higher CMR during COVID-19 period of 18.9, (95% CI 17.2–20.8) per 1000 person compared to 12.5 (95% CI 11.6–13.4) per 1000 person years before COVID-19. The risk of dying among children under 5 years compared to those aged between 5 and 14 years was higher during the COVID-19 pandemic period (aHR = 18.0, 95% CI 13.6–24.0) than pre-pandemic (aHR = 10.4, 95% CI 8.8–12.3).

**Conclusion:**

The COVID-19 pandemic increased all-cause mortality in the Iganga Mayuge HDSS population cohort in Eastern Uganda, particularly among children under five, likely due to restricted healthcare access and economic disruptions. Pandemic response measures should prioritize vulnerable populations at higher risk of malnutrition and preventable diseases to mitigate future negative impacts.

## Introduction

Research on the Coronavirus Disease (COVID-19) pandemic has largely focused on direct deaths, yet indirect mortality driven by healthcare disruptions, economic hardship, and behavioral changes is poorly quantified. This gap obscures the true toll of the pandemic. Understanding the overall effect of COVID-19 pandemic on mortality is essential for informing policies on mitigating its impact and designing more effective response mechanisms to future pandemics. By June 2021, more than a year after the World Health Organization declared the COVID-19 outbreak a pandemic, nearly 4,000,000 deaths had been reported [1]. In settings where excess mortality has been analyzed, the total mortality is larger than the reported number of COVID-19-related deaths [2–5]. While the gap between excess mortality and officially reported COVID-19-related deaths is partly explained by under-reporting, previous outbreaks have demonstrated that indirect health effects caused by reductions in the delivery of routine health services could be as important as the direct consequences [6]. The threat of this double crisis is particularly worrying for countries which are faced with poor mortality surveillance systems and Civil Registration and Vital Statistics (CRVS) for correctly monitoring deaths and other population indicators. Low and Middle Income Countries (LMICs) have higher mortality rates, more fragile health systems, and health outcomes that are more sensitive to income shocks, such as those unleashed by the COVID-19 pandemic [6, 7]. These factors heighten the risk of short-term downturns in the utilization of preventive, promotive, and curative care to erode the hard-fought progress towards reducing mortality.

Excess mortality during the COVID-19 pandemic was the combination of deaths caused, or contributed to, by infection with Severe Acute Respiratory Syndrome Coronavirus 2 (SARS-CoV-2) plus deaths that resulted from the widespread changes in socioeconomics, social behaviors, healthcare seeking patterns that accompanied non-pharmaceutical interventions as responses to the emergency [8, 9]. In other words, it was a measure of the overall impact of COVID-19 on the population [10].

In the first months of the 2020 pandemic, it is very likely that attribution of deaths to COVID-19 pandemic was unreliable because of the novelty of the virus, uncertainty about its precise clinical manifestations, and an absence of widespread testing for laboratory confirmation of infection. The problem of acknowledgement of cause was further complicated by the probability that some deaths might have occurred as a result of indirect effects, such as reduced diagnosis and care for non-COVID conditions, plus the broader effect of voluntary and obligatory behavioral change such as social distancing [11].

Uganda experienced three waves of COVID-19 pandemic. The first wave was in mid to late 2020, second wave in April –August 2021, and the third wave in December 2021–February 2022. Each of these waves was characterized by predominantly different SARS-COV-2 variants namely the Alpha, Delta, and Omicron variants respectively. The mortality rates were different in each of the three waves, with Delta having the highest, followed by Omicron and Alpha variants [12].

The Ugandan government responded with non-pharmaceutical interventions like restricting population movement by closing borders, reducing public transport, halting non-essential activities, and issuing shelter-in-place orders. These restrictions negatively affected healthcare seeking patterns, the economy where people lost income, led to increased prices, over-burdened social safety nets which pushed vulnerable groups further into poverty and increased financial and other barriers. Movement restrictions reduced physical access, exacerbated by reduced transport availability and the real or perceived threat of prosecution for travelling in public spaces [13].

One approach that has been used to avoid the challenge of analyzing cause-specific mortality was to assess the impact of the pandemic on total excess deaths [14]. Total excess mortality captures both the direct and indirect impacts of COVID-19 on mortality and is not affected by validity of cause of death [11].

Understanding the overall impact of the COVID-19 pandemic on mortality in countries like Uganda, is challenging because of weaknesses in mortality surveillance and CRVS systems. This limitation obscures the true burden of the pandemic and the direct or indirect effects. The different waves of COVID-19 in Uganda were characterised by stringent non-pharmaceutical interventions, disruptions of access to healthcare, limited economic activity, and social behaviors that could have contributed to additional mortality. There is limited research that has explored how these indirect effects impacted vulnerable populations like children and the elderly.

## Uganda’s health system and the COVID-19 pandemic containment and response

Uganda’s healthcare system faced challenges during the COVID-19 pandemic due to pre-existing weaknesses in infrastructure, resource allocation, and access to care. The country’s health system, already strained by a high burden of infectious diseases (e.g., HIV, malaria) and a growing incidence of non-communicable diseases (NCDs) [15], struggled to manage the dual demands of pandemic response and routine service delivery. Public health interventions, such as lockdowns and movement restrictions, disrupted access to essential services like antenatal care, immunizations, and treatment for chronic conditions [16, 17].

**Fig. 1:**
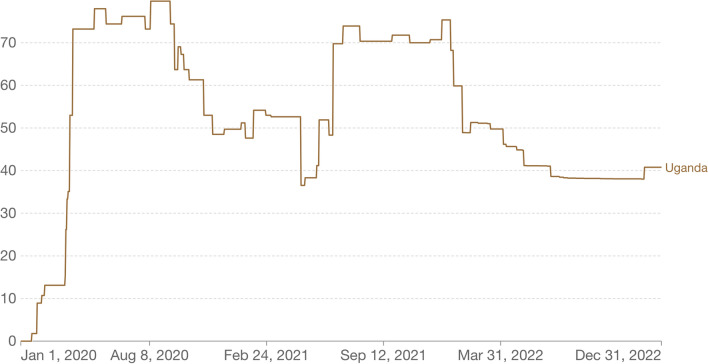
The Uganda Temporal trajectories of the OxCGRT stringency index. Source: Oxford COVID-19 Government response tracker, blavatnik school of government, university of oxford – Last updated24 July 2023 OurWorldInData.org/coronavirus [18] (https://ourworldindata.org/covid-stringency-index)

The COVID-19 pandemic officially reached Uganda on March 21, 2020, when the first case was confirmed. In response to the rising threat, the Ugandan government swiftly implemented a series of non-pharmaceutical measures to curb the spread of the virus. A nationwide lockdown was announced effective March 23, 2020, which included the closure of schools, public transportation, and non-essential businesses, imposed travel restrictions, suspended public gatherings, and enforced a curfew. The *Oxford COVID-19 Government Response Tracker* (OxCGRT) Containment and Health Index (https://ourworldindata.org/covid-stringency-index) is shown in Fig. 1. This is a composite measure of thirteen of the response metrics and the index on any given day calculated as the mean score of the eleven metrics, each taking a value between 0 and 100 [100 = strictest). This index builds on the Stringency Index, using its nine indicators plus testing policy, the extent of contact tracing, requirements to wear face coverings, and policies around vaccine rollout. It is calculated based on the following thirteen metrics: school closures; workplace closures; cancellation of public events; restrictions on public gatherings; closures of public transport; stay-at-home requirements; public information campaigns; restrictions on internal movements; international travel controls; testing policy; extent of contact tracing; face coverings; and vaccine policy. The full description of how this index is calculated can be found here https://github.com/OxCGRT/covid-policy-tracker/blob/master/documentation/index_methodology.md.

To fill these gaps, we examined excess mortality during the pandemic and identified its effects on all-cause mortality across demographic groups. We quantified the impact of the COVID-19 pandemic on overall mortality using longitudinal data to assess trends over time and evaluate which populations were most affected.

This study assesses the impact of COVID-19 on all-cause mortality in Eastern Uganda, using longitudinal data from the Iganga Mayuge Health and Demographic Surveillance Site (IMHDSS) from 2015 to 2021. We quantify excess mortality across demographic groups, with a focus on children under five, to inform equitable pandemic response strategies in resource-limited settings.

## Methods and materials

### Design

This was a retrospective analysis of data from a longitudinal population cohort using the demographic events and mortality surveillance information collected from 2015 to 2021. This design leveraged the existing population cohort of IMHDSS that was established in 2005 as a community-based research and research training platform under Makerere University Centre for Health and Population Research (MUCHAP). It makes it possible to examine historical trends in mortality and demographic events over an extended period. Using previously collected data, mitigated the possible challenges of conducting a prospective cohort study, particularly during a pandemic when real-time data collection was constrained by movement restrictions and resource limitations. The retrospective analysis enabled the evaluation of mortality patterns before and during the pandemic, facilitating comparisons that highlight excess mortality attributable to direct and indirect effects of COVID-19.

### Setting and participants

**Fig. 2:**
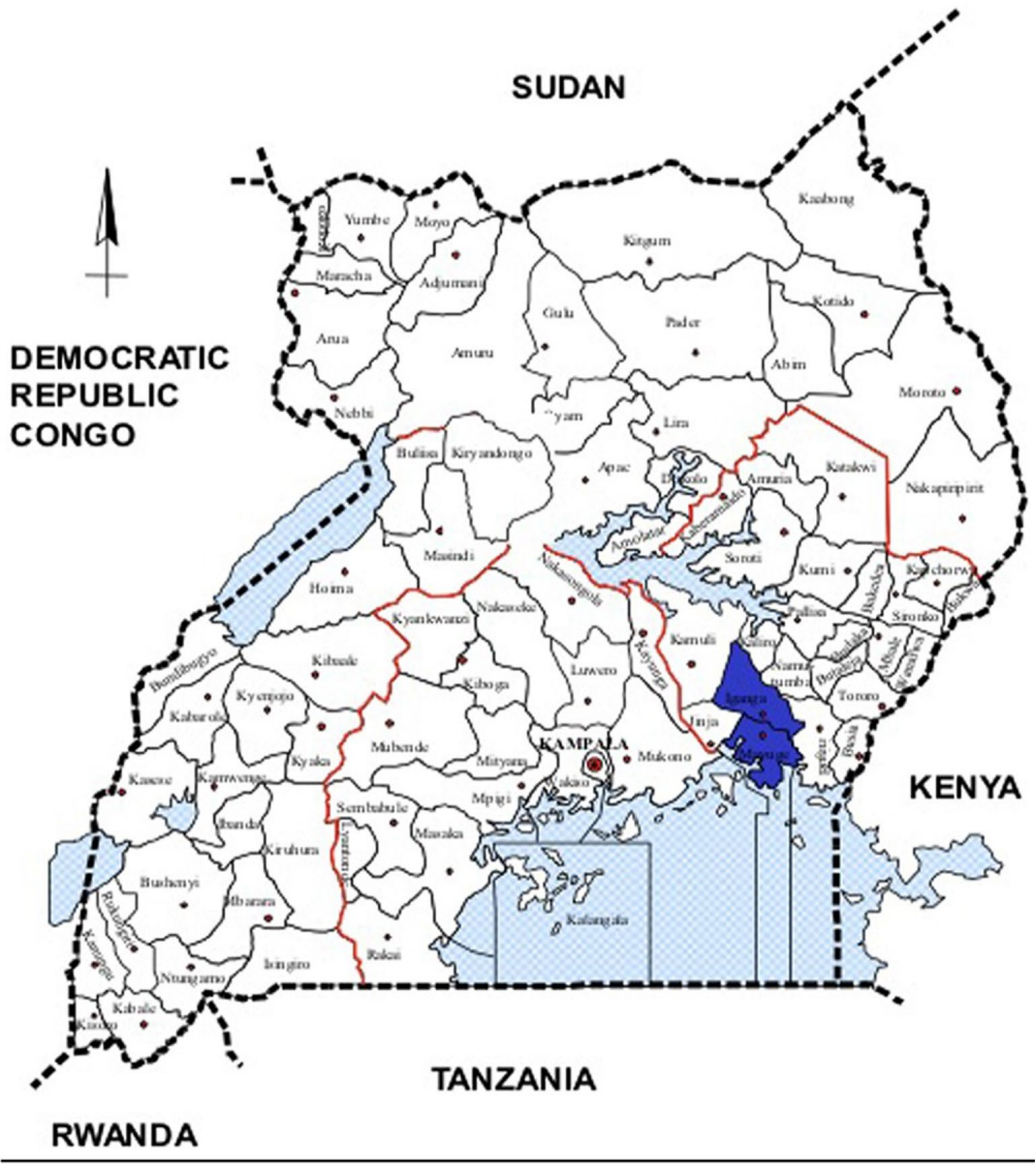
The shaded area shows the districts where Iganga mayuge health and demographic surveillance site (IMHDSS) is located

The IMHDSS located in Iganga and Mayuge districts of Eastern Uganda as shown in Fig. 2, covers a total of 65 villages with 101,927 individuals living in about 18,000 households under surveillance. The demographic surveillance area is estimated to be 155 Km^2^. IMHDSS conducts census-level data collection on key demographic events of births, deaths, pregnancies, and their outcomes, and in-and out-migrations. Death reporting between the HDSS census rounds is enhanced by a network of village heath team members who are resident members of the community. The population is predominantly of Muslim religion (53%), 51% of residents are females, the median age is 22.5 years and 60% live in rural areas. The main source of income for the rural population is farming/agriculture (51%) whereas it is shop/business for the peri-urban population. The IMHDSS community is served by 16 community-based lower-level health centres (HC) (HC III and HC II) and two hospitals (one district referral public hospital in Iganga and one faith-based hospital in Mayuge district) [19].

### Data sources and mortality measurement/surveillance

The Iganga Mayuge health and demographic surveillance site (IMHDSS) conducts annual census of all households in the demographic surveillance area to update data on demographic events of births, deaths, migration socio-economic status, and education [19]. A team of trained and experienced data collectors visit households to update all the events. Data collection is done electronically using handheld tablets computers.

The HDSS implements an elaborate mortality surveillance system where, in addition to the HDSS periodic update rounds, there is a network of community-based scouts composed village heath team members, local council chairpersons and other opinion leaders who report all deaths occurring in households before the HDSS update round is conducted. The scouts were jointly identified by community members and the HDSS leadership with the responsibility of reporting to the HDSS all deaths and births as they occur in their villages of residence in near real time. This death notification information is used to conduct verbal autopsies that are used to determine the probable cause of death by a trained and dedicated physician (medical doctor).

### Data harmonisation process

This study is part of a large initiative aimed at quantifying the impact of the COVID-19 pandemic on mortality in various rural and urban settings in sub-Saharan Africa and South Asia, supported by continuous health and demographic surveillance systems (HDSSs) [20] (submitted to the Lancet for publication). Although enrolling individuals in defined areas and systematically updating vital events is common across all HDSS platforms, the raw data are collected and stored in different formats and structures. Therefore, the data were cleaned, and harmonized using established principles and guidelines [21, 22]. The data preparation and harmonization process were further guided by the INDEPTH Network I-Share data harmonization and quality assurance process [23].

### Statistical analysis

A time series dataset was constructed from the HDSS population data that included all deaths registered. Number of deaths and person years at risk were counted for each quarter of the year from January 2015 to December 2021. Each quarter was classified as “pre-pandemic” (before January 2020), and “during pandemic” (January 2020 to December 2021). Other sociodemographic variables included in the analysis were education level, area of residence, household size and wealth quintile.

All these sociodemographic variables are updated at least once a year as part of the HDSS census. Individuals' ages were grouped using WHO standard age groups: 0–4, 5–14, 15–49, 50–64, and 65 + years. This grouping helps in understanding trends and impacts across different life stages, such as early childhood, school age, working age, and older adulthood. The number of people in a household was also categorized using groups of 1–3, 4–6, 7–10, and 11+ individuals. This was chosen to maintain consistency in size across all household groups.

Household wealth Quintile (a proxy for social economic status) was estimated with an asset-based index that combines information about ownership of consumer goods, construction materials in the main dwelling; type of toilet facilities and sources of water; sources of energy; ownership of modern assets; and livestock using principal components analysis [24]. As such, each household's wealth Quintile score represents its position in the wealth distribution relative to other households within the HDSS. The wealth index was divided into household wealth quintiles, in which the first quintile represented the poorest households and the fifth the richest household.

### Descriptive statistics and crude mortality rates (CMR)

Frequencies for age group, sex, education level, household size and household wealth quintile were generated. The Crude Mortality Rates (CMR) is a demographic measure used to estimate the overall mortality rate within a specific population over a defined period. It is calculated by dividing the number of deaths by person years. The person-years represent the sum of the time each individual was exposed to the risk of death during the study period, and it is computed using the formula below.

$$\begin{aligned} Person - Years & = Population\ at\ Risk \\ & \quad \times Time\ under\ Observation \end{aligned}$$

where "Population at Risk" refers to the number of individuals in the population at the midpoint of the time interval, and "Time under Observation" represents the length of time each individual was part of the population during the study period.

The total number of deaths that occurred within the study population during the defined time period, was used to calculate the Crude Mortality Rate using the formula below:

$$\begin{aligned} CMR = &\left( Total\,Number\,of\,Deaths/ \right. \\ & \quad \left. Total\,Person - Years \right) \times 1000 \end{aligned}$$

The CMR result was expressed as the number of deaths per 1000 person-years and the respective confidence intervals were reported. The CMR was computed and accounted for variations in population size and follow-up time. This rate provides an estimate of mortality within the population under study, accounting for both the size of the population and the duration of observation.

### Multivariate analysis

Time series models and the Cox Proportional Hazard model was used to analyze mortality trends and determine the impact of COVID-19 on all-cause mortality. The combination of time series analysis and the Cox Proportional Hazard model provided a robust approach to understanding mortality patterns. Time series models quantified excess deaths over time, while the Cox model identified socioeconomic and demographic risk factors influencing mortality. Model assumptions were rigorously tested to ensure validity, and appropriate corrective measures were applied when necessary.

### Cox proportional hazard model

A Cox Proportional Hazard model was used to estimate the Hazard ratio associated with death for each socio-economic and demographic characteristic. Socio-demographic characteristics included age group, sex, area of residence, household size, highest level of education attained and wealth quintile. We tested the critical proportional hazards assumption using Schoenfeld residuals and examined interactions between pandemic periods and demographic characteristics to ensure model validity. Variance Inflation Factors (VIFs) were used to assess multicollinearity, ensuring that predictor variables were not highly correlated and that the model remained robust. Interaction terms, particularly between pandemic periods and demographic characteristics, were tested to ensure model accuracy. We assessed interactions of each predictor variable between the—“pre-pandemic” (2015–2019) and “during pandemic” (2020–2021) periods.

### Time series model

This model was used to locate the exact time when excess deaths occurred. Excess deaths were determined by, first, establishing a counterfactual estimate of the number of deaths by sex, age and other socio-economic and demographic factors. Because of small sample size, mortality rates are computed on quarterly aggregated data instead of monthly or weekly to prevent singularities. Let ${D}_{s,t}$ be the number of deaths at time $t$ (given in 3 months intervall). Let ${year}_{t}$ be the categorical year index in which months grouped in quarters occurred, ${quater}_{t}$ be 3 months period, and $Age$ be the age of participants aggregated in 10-year age groups. We fitted a quasi-Poisson generalized additive model on all available mortality data from 2015 to 2019. Quasi-Poisson model was chosen over Poisson regression due to its ability to adapt to over-dispersion in count data, providing more reliable standard errors, and its flexibility in modeling non-linear trends through smooth functions.

The general form of generalized additive model is.

$$\begin{aligned} \log \left( {\mu_{s,t} } \right) & = f_{s}^{1} \left( {year_{t} } \right) \otimes f_{s}^{2} \left( {quater_{t} } \right) \\ & \quad + \beta_{0,s} + \beta_{1,s} { }Age{ } \end{aligned}$$

where, ${\mu }_{s,t}=E\left(\frac{{ D}_{s,t}}{{PM}_{s,t}}\right|t,{quater}_{t},{year}_{t},Age). {PM}_{s,t}$ is the total exposure measured in person month for a particular age group, $f_{s}^{1}$ and $f_{s}^{2}$ are smooth functions of time $t$ which account for within-year and within quarter seasonal variation and ⨂ is a tensor product between smooth functions. The yearly trend ${f}_{s}^{1}$ is modeled with a thin-plate spline and within-quarter variation ${f}_{s}^{2}$ is fitted using a penalized cyclic cubic regression spline. ${\beta }_{0}$ are intercepts and ${\beta }_{1}$ are regression coefficients. Since we modelled the crude mortality rate, the offset is included in left side term of the equation. Assumptions such as the independence of errors were checked using autocorrelation function plots of the residuals, while the degree of smoothing was selected using cross-validation to avoid overfitting.

Model fit was assessed using the Akaike Information Criterion (AIC) and residual plots while the stability of seasonal and long-term trends was verified by comparing models with different combinations of polynomial and spline-based terms. The final model is used to obtain predictions of the expected deaths for all $t$ in 2020 and 2021, with both a point estimate and a standard error being produced. We calculated absolute excess and relative excess deaths as follows:

$$\begin{aligned} Absolute\,excess\,deaths\, & = \,Observed\,deaths\, \\ & \quad - \,Expected\,deaths \end{aligned}$$

$$\begin{aligned} Relative\,excess\,deaths\, & = \,Observed\,deaths/\\ & \quad Expected\,deaths \end{aligned} $$

All analyses were performed using R software (R version 4.1.1).

## Results

From 2015 to 2021, a total of 132,367 individuals who reside in the Iganga Mayuge HDSS were followed up with a combined 615,790 person years. In the same period, 3,424 deaths were registered through the HDSS household update census surveys and continuous notification of death by community-based scouts [19].

### Crude mortality rate (CMR)

**Fig. 3:**
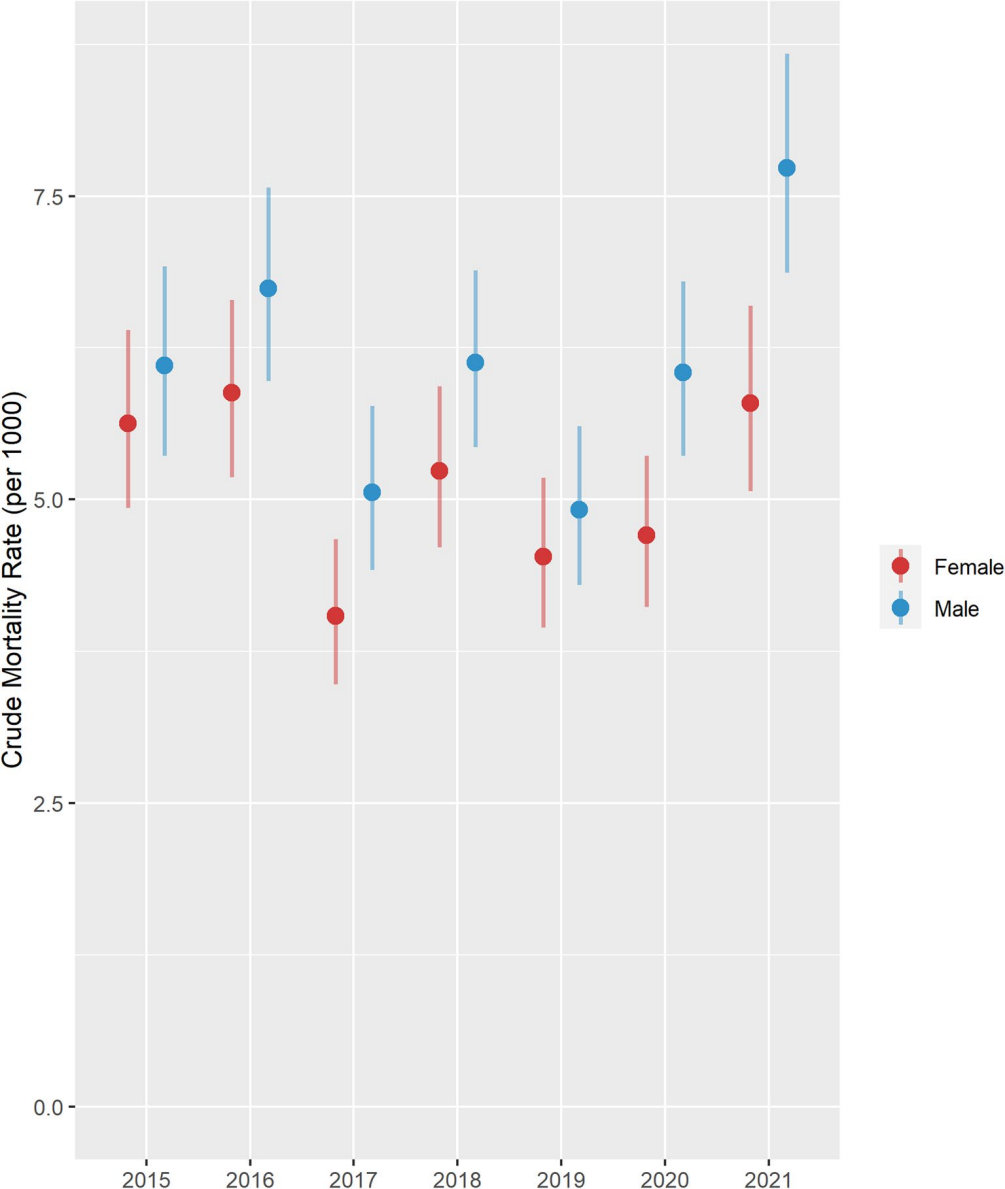
Crude Mortality per 1000 person years by sex, 2015–2021

The Crude Mortality rate has fluctuated over time since 2015 and has been consistently high for males compared to females. The lowest CMR was recorded in 2017 for females while it was lowest for males in 2019. During the pandemic period (2020–2021), there was a significantly higher CMR for males compared to females. In 2020, CMR was 6.0 per 1000 person years for males versus 4.7 per 1000 person years for females) and in 2021, it was7.7 per 1000 person years for males versus 5.8 per 1000 person years for females as shown in Fig. 3.

### Crude mortality rates and socio-demographic characteristics

Crude mortality rate was computed per 1000 person years for selected socio-demographic characteristics as shown in Table 1. Overall, there was a higher CMR (6.0, CI [5.6–6.3] per 1000 person years) during COVID-19 period compared to before COVID-19 (5.4, CI [5.2–5.6] per 1000 person years) (Table 1). CMR of older members (65 years and above) of this population cohort was high both before and during COVID-19 pandemic periods. However, children under 5 years had a significantly higher CMR during COVID period of 18.9 per 1000 person years (95% CI 17.2–20.8) compared to 12.5 per 1000 person years (95% CI 11.6–13.4) before the COVID-19 pandemic.

**Table 1:** Deaths and Crude death rates (CDR) before COVID-19 and during COVID-19 period

Variable	Before COVID-19						During COVID-19				
	Category	Person years	Deaths	CDR	CDR lower CI	CDR upper CI	Person years	Deaths	CDR	CDR lower CI	CDR upper CI
Overall		443792	2397	5.4	5.2	5.6	171999	1027	6.0	5.6	6.3
Sex	Female	226025	1141	5.0	4.8	5.3	87905	456	5.2	4.7	5.7
	Male	217767	1256	5.8	5.5	6.1	84095	571	6.8	6.2	7.4
WHO age group	0–4	61334	765	12.5	11.6	13.4	22647	429	18.9	17.2	20.8
	5–14	137237	163	1.2	1.0	1.4	50844	53	1.0	0.8	1.4
	15–49	211302	554	2.6	2.4	2.8	83888	194	2.3	2.0	2.7
	50–64	22449	274	12.2	10.8	13.7	10007	87	8.7	7.0	10.7
	65 +	11469	641	55.9	51.6	60.4	4614	264	57.2	50.5	64.6
Education level	None	96165	487	5.1	4.6	5.5	37279	187	5.0	4.3	5.8
	Primary	237067	1328	5.6	5.3	5.9	91772	598	6.5	6.0	7.1
	Secondary	79088	426	5.4	4.9	5.9	30455	177	5.8	5.0	6.7
	Post-Secondary	30862	152	4.9	4.2	5.8	12243	65	5.3	4.1	6.8
Residence	Rural	253880	1534	6.0	5.7	6.4	99194	704	7.1	6.6	7.6
	Urban	189911	863	4.5	4.2	4.9	72805	323	4.4	4.0	4.9
Household size	1–3	97155	559	5.8	5.3	6.3	37479	238	6.4	5.6	7.2
	4–6	118445	679	5.7	5.3	6.2	46393	273	5.9	5.2	6.6
	7–10	136722	712	5.2	4.8	5.6	52732	295	5.6	5.0	6.3
	11 +	91470	447	4.9	4.4	5.4	35395	221	6.2	5.4	7.1
Social economic status	1(Poorest)	88534	524	5.9	5.4	6.4	34647	288	8.3	7.4	9.3
	2	85041	525	6.2	5.7	6.7	33133	208	6.3	5.5	7.2
	3	89864	508	5.7	5.2	6.2	34947	211	6.0	5.3	6.9
	4	96167	495	5.1	4.7	5.6	37108	188	5.1	4.4	5.8
	5(Richest)	84186	345	4.1	3.7	4.6	32164	132	4.1	3.4	4.9

Rural residents had a higher CMR during the pandemic period at 7.1 per 1000 person years (95% CI 6.6–7.6) compared to 6.0 per 1000 person years, (96% CI 5.7–6.4) pre-pandemic. CMR was higher amongst males than females in this population cohort before COVID-19 pandemic however, it was significantly higher during the COVID-19 period (CMR = 6.8, 95% CI 6.2–7.4) compared to the period before COVID-19 (CMR = 5.8, 95% CI 5.5–6.1). During the COVID-19 period, a significantly higher CMR was observed among people whose highest level of education was primary (CMR = 6.5 per 1000 person years, CI 6.0–7.1), compared to pre-pandemic (CMR = 5.6 per 1000 Person years, CI 5.3–5.9).

Larger households had a higher risk of mortality during the COVID-19 period with CMR significantly higher amongst residents from households with 11 or more individuals (CMR = 6.2, 95% CI 5.4–7.1) during COVID-19 period compared to 4.9 (95% CI 4.4–5.4) observed in the period before COVID-19. Similarly, the CMR for individuals that came from poorest households was significantly higher during the COVID-19 period (CMR = 6.2, 95% CI 5.4–7.1) compared to the period before COVID-19 (CMR = 4.9, 95% CI 4.4–5.4).

### Hazard ratios of dying in the different socio-demographic groups

**Fig. 4:**
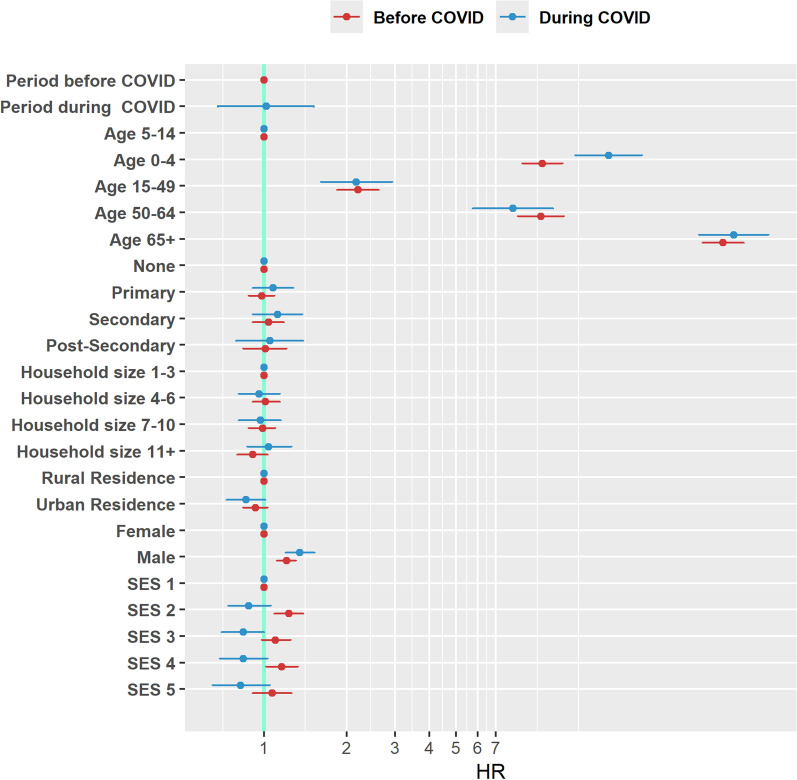
This shows the Hazard ratios (risk of dying) before and during the COVID period by different factor variables

Male participants had 40% (HR = 1.4, CI 1.2–1.5) greater risk of dying during COVID, and a 20% (HR = 1.2, CI 1.1–1.3) greater risk of dying before COVID compared to the female participants (Fig. 4). Old individuals (65 years and above) had a higher risk of dying in this population cohort irrespective of the COVID-19 pandemic. In children under five years, the risk of dying was significantly higher during the COVID-19 period (HR = 18.0 (95% CI 13.6–24.0) than before (HR = 10.4 (95% CI 8.8–12.3)) compared to their counterparts aged 5–14 years. A similar pattern of higher risk of dying during COVID-19 period is seen in members of households whose wealth quintile is poorer compared to poorest.

### Excess mortality during the COVID-19 period

**Fig. 5:**
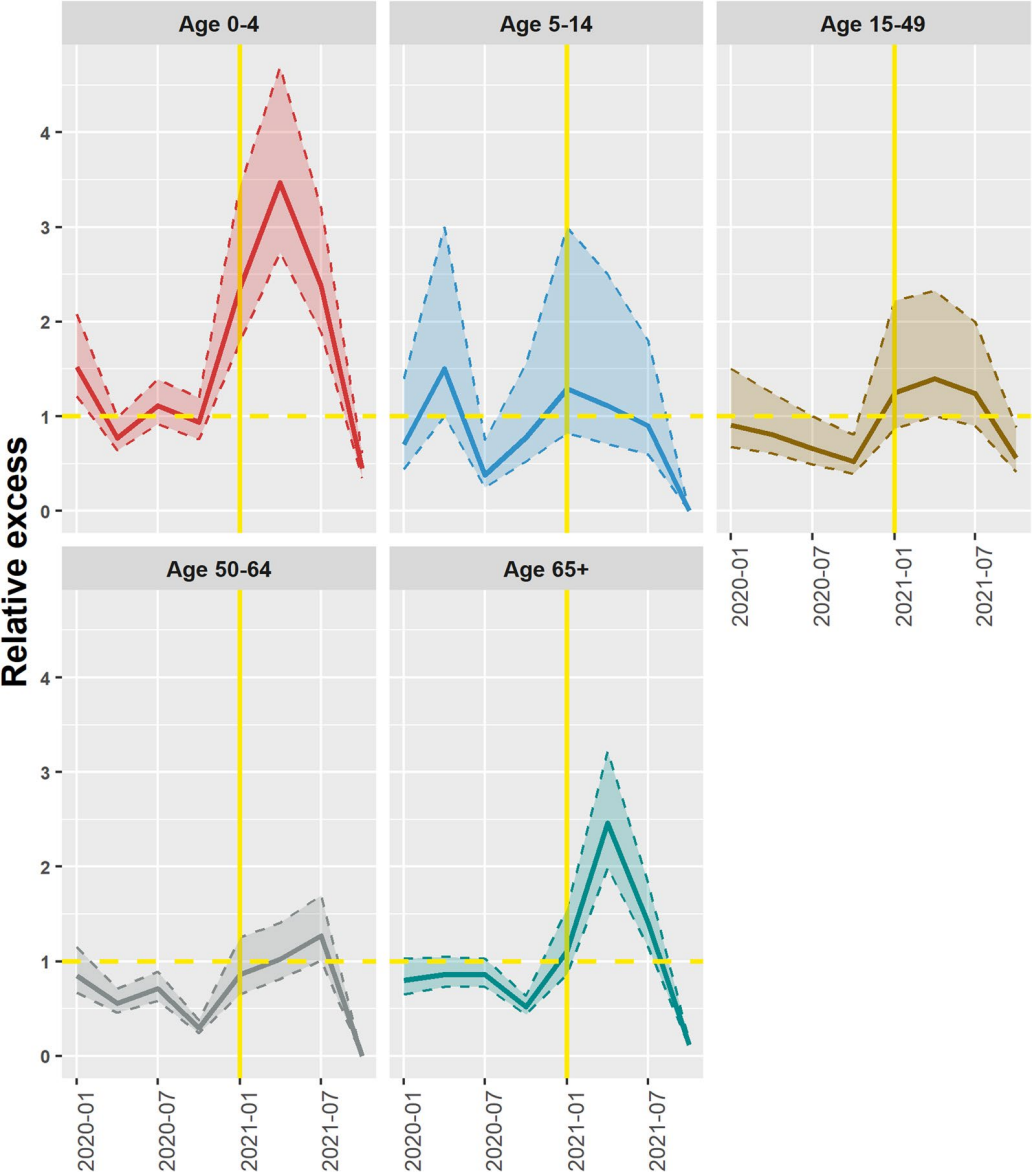
Relative excess deaths by age group. The Y-axis represents the relative excess deaths for example 1.5 means 50% more deaths compared to what was expected

Excess mortality was observed during the COVID-19 period with mortality observed in the 0–4 years age group (4.7 in 2021), followed by the 65 and above age group (3.2 in 2021). The 50–64 age group registered the lowest peak of excess mortality at 1.7 in the second quarter of 2021 (Fig. 5). Across all age groups, excess mortality was highest around the first and second quarters of 2021.

## Discussion

The findings of this analysis highlight important shifts in mortality trends within the Iganga Mayuge HDSS population cohort, particularly during the COVID-19 pandemic. This analysis of all-cause mortality illustrates that the pandemic led to excess deaths beyond the expected. By comparing mortality data from the pre-pandemic (2015–2019) and pandemic (2020–2021) periods, we identified increases in mortality across demographic groups, with vulnerable populations, including children and the elderly, being particularly affected.

From 2015 to 2021, the crude mortality rate (CMR) was consistently higher among males compared to females, with a marked increase observed in 2020 and 2021. During the pandemic, the CMR for males reached 7.7 per 1,000 person-years, compared to 5.8 for females. Our findings are different from [25] who showed that variations in excess mortality over the period examined were similar for both men and women and that there were no significant differences between genders. On the other hand findings from this analysis agree with several studies that have shown that men were more likely to die during the COVID-19 pandemic [26–29]. These differences may be partially explained by differences in risk exposures and health-seeking behaviors between genders, as well as potential socioeconomic and healthcare access disparities exacerbated by the pandemic. The heightened male mortality rate during the pandemic underscores the need for gender-sensitive health policies, especially in emergency response efforts.

The analysis reveals that, in age-specific mortality analysis, children under five years and adults aged 65 and above experienced significantly higher mortality during the pandemic. The CMR for children under five surged from 12.5 per 1,000 person-years pre-pandemic to 18.9 during the pandemic. Similarly, the oldest age group showed persistently high CMRs throughout the period under review, with additional increases observed during the pandemic. Hanifi and colleagues also found 28% increase in excess deaths in older people in 2020 compared to previous 5 years using data from Matlab Health and Demographic Surveillance [1]. These findings suggest that young children and older adults were particularly vulnerable to the indirect effects of the pandemic, such as reduced healthcare access due to movement restrictions and strained health systems. The elevated hazard ratios for these age groups highlight a critical gap in protecting the vulnerable and most susceptible populations during health crises. Childhood illnesses are of acute nature and needed immediate attention. Pneumonia and Malaria are among the leading causes of mortality in children [30] which need critical attention within a few hours, beyond which conditions can be fatal. However, these excess deaths can be considered a part of the COVID-19 pandemic burden as they would not have occurred in its absence [31].

The analysis also revealed disparities in mortality based on education level, residence, and socioeconomic status. Individuals with only primary education, rural residents, and those from poorer households experienced higher mortality rates, which worsened during the pandemic. For example, rural residents had a CMR of 7.1 per 1,000 person-years during the pandemic, compared to 6.0 pre-pandemic, while the CMR for individuals from the poorest households increased from 5.9 to 8.3. These trends align with findings from South Africa, where excess mortality was disproportionately higher in low-income communities, largely due to limited healthcare access and higher infection risks [32]. Similar patterns were observed in Bangladesh, where pre-existing health disparities intensified during the pandemic, leading to higher death rates among the socioeconomically disadvantaged [33]. These socioeconomic gradients in mortality underscore the need for inclusive public health responses that prioritize vulnerable populations, ensuring equitable access to healthcare and social protection measures during times of crisis.

The government of Uganda implemented stringent non-pharmaceutical interventions to control the spread of the virus, such as travel restrictions, lockdowns, and the closure of essential services [34] and evidenced by the higher OxCGRT stringency index [27]. While these measures aimed to prevent COVID-19 infections, they also disrupted access to healthcare, impacted income generation, and affected daily activities, which in turn amplified health risks, particularly among those with limited financial and social resources. This is similar to what was observed in research from Thailand, Malaysia, Italy, and the UK which revealed disruptions to healthcare access by NPIs [35]. Our findings suggest that indirect effects, like delayed healthcare, poverty, and isolation, exacerbated the pandemic's impact on mortality, underscoring the multifaceted nature of excess deaths observed during this period.

There are limitations in this analysis that should be acknowledged. First, HDSS datasets, given their large scale and extensive population coverage, often exhibit lower data quality than data collected through more active methods like clinical trials or smaller cohort studies. Furthermore, ensuring consistent data collection across time points is essential for reliable time-trend analyses. Within the IMHDSS, the frequency of data collection rounds changed over the study period. Notably, in 2020 and 2021, the IMHDSS team was unable to conduct any update rounds due to COVID-19 lockdowns and movement restrictions, which limited household visits. During this period, the Uganda National Council for Science and Technology also imposed a temporary suspension on research activities involving in-person interviews. This interruption differed significantly from the usual biannual or triannual data collection schedule, potentially resulting in missed records, especially infant deaths, due to lack of home visits.

## Conclusions

In conclusion, there was increased all-cause mortality in specific demographic groups that can be attributed to the indirect impact of COVID-19 pandemic on public health in Uganda. The pandemic worsened existing vulnerabilities, particularly among children under five, older adults, males, rural residents, and individuals from lower socioeconomic backgrounds. These findings contribute to efforts exploring the multifaceted nature of excess mortality during the pandemic. Pandemic responses negatively affected healthcare access, amplified poverty, and increased social isolation as was reported in other low- and middle-income countries (LMICs). Future pandemic response strategies should focus on safeguarding healthcare access for vulnerable populations, with a priority on preventive and supportive services tailored to high-risk groups. Targeted interventions, such as income support and improved healthcare accessibility in rural areas, could help mitigate these disparities. Such measures will not only safeguard healthcare access for vulnerable populations during crises but also inform tailored preventive and supportive services for high-risk groups. Ongoing mortality surveillance is crucial, particularly through the establishment of country-wide sample-based mortality surveillance systems that can leverage methods like the HDSS to report community deaths and verbal autopsy to determine their probable causes of death.

## Data Availability

Data for computing mortality indicators by year, age and sex are available from the MRC/Wits Agincourt Research Unit Data Repository (https://data.agincourt.co.za/index.php/catalog/347). Data containing other covariates used in the analysis reported in this manuscript can be accessed through a formal request to the corresponding author.

## Abbreviations

COVID-19: Coronavirus disease
CRVS: Civil registration and vital statistics
LMICs: Low- and middle-income countries
SARS-CoV-2: Severe acute respiratory syndrome coronavirus 2
HIV: Human immunodeficiency virus
NCD: Non-communicable diseases
OxCGRT: Oxford COVID-19 government response tracker
IMHDSS: Iganga mayuge health and demographic surveillance site
MUCHAP: Makerere university centre for health and population research
HC: Health Centre
HDSS: Health and demographic surveillance system
INDEPTH: International network for the continuous demographic evaluation of population health
WHO: World health organisation
CMR: Crude mortality rate
VIF: Variance inflation factor
AIC: Akaike information criterion
